# Toward Sustainable and Comprehensive Control of Schistosomiasis in China: Lessons from Sichuan

**DOI:** 10.1371/journal.pntd.0001372

**Published:** 2011-10-25

**Authors:** Edmund Y. W. Seto, Justin V. Remais, Elizabeth J. Carlton, Shuo Wang, Song Liang, Paul J. Brindley, Dongchuan Qiu, Robert C. Spear, Long-De Wang, Tian-Ping Wang, Hong-Gen Chen, Xing-Qi Dong, Li-Ying Wang, Yang Hao, Robert Bergquist, Xiao-Nong Zhou

**Affiliations:** 1 School of Public Health, University of California, Berkeley, California, United States of America; 2 Rollins School of Public Health, Emory University, Atlanta, Georgia, United States of America; 3 College of Public Health, The Ohio State University, Columbus, Ohio, United States of America; 4 Department of Microbiology, Immunology & Tropical Medicine, George Washington University Medical Center, Washington, D.C., United States of America; 5 Institute of Parasitic Diseases, Sichuan Center for Disease Control and Prevention, Chengdu, People's Republic of China; 6 School of Public Health, Peking University, Beijing, People's Republic of China; 7 Anhui Institute of Parasitic Diseases, Wuhu, People's Republic of China; 8 Jiangxi Institute of Parasitic Diseases, Nanchang, People's Republic of China; 9 Yunnan Institute of Endemic Diseases, Dali, People's Republic of China; 10 Ministry of Health, Beijing, People's Republic of China; 11 Ingerod, Brastad, Sweden; 12 National Institute of Parasitic Diseases, Chinese Center for Disease Control and Prevention, Shanghai, People's Republic of China; Swiss Tropical and Public Health Institute, Switzerland

## Abstract

Triggered by a fascinating publication in the *New England Journal of Medicine* detailing China's new multi-pronged strategy to control and eventually interrupt the transmission of *Schistosoma japonicum*, this *PLoS Neglected Tropical Diseases* Debate critically examines the generalizability and financial costs of the studies presented from the marshlands of the lake region. Edmund Seto from the University of California and colleagues emphasize that the epidemiology and control of schistosomiasis varies according to the social-ecological context. They conjecture that the successful intervention packages piloted in the lake region is not fully fit for the hilly and mountainous environments in Sichuan and Yunnan provinces, and hence call for more flexible, setting-specific, and less expensive control strategies. In response, Xiao-Nong Zhou from the National Institute of Parasitic Diseases at the Chinese Center of Disease Control and Prevention and colleagues explain the steps from designing pilot studies to the articulation and implementation of a new national control strategy through a careful process of scaling-up and adaptations. Finally, the two opponents converge. The need for integrated, intersectoral, and setting-specific control measures is stressed, supported by rigorous surveillance and continuous research. Experiences and lessons from China are important for shaping the schistosomiasis elimination agenda.

## Viewpoint by Edmund Seto, Justin Remais, Elizabeth Carlton, Shuo Wang, Song Liang, Paul Brindley, Dongchuan Qiu, and Robert Spear: Toward Sustainable and Comprehensive Control of Schistosomiasis in China: Lessons from Sichuan

Over the five past decades, China has made great strides toward reducing the prevalence of schistosomiasis japonica, largely through a strategy based on chemotherapy and snail control [1]. Yet, progress toward further reduction in prevalence and disease elimination has been difficult due to high rates of reinfection. This has resulted in the State Council of China's establishment of two aggressive control targets in 2004: first, that the rate of infection in humans in all endemic counties be less than 5% by 2008, and second, that by 2015, the rate be less than 1% [2], which has accelerated the translation of research findings into new strategic plans for sustainable control.

Recent findings by Wang et al. [2] from an intervention study conducted in the lake region of China have lead to a new national control strategy that emphasizes a comprehensive approach for combating schistosomiasis japonica that goes beyond drug therapy programs and includes elements of cattle removal, sanitation provision, environmental management, and health education. In the authors' words, “as a result of these data, the Chinese government has adopted the interventions used in [the] study as the national strategy for the control of schistosomiasis [the recently introduced Schistosomiasis Prevention and Control Regulations].” Moreover, they state that despite methodological limitations, they “believe that the new national strategy can substantially reduce the burden of schistosomiasis in China”.

While we applaud comprehensive disease control approaches, we feel that two points are worthy of discussion and debate: generalizability and cost. First, we question the generalizability of this intervention study, conducted in four villages in the lakes region, as the environmental conditions and epidemiology of schistosomiasis transmission vary greatly across China [1]. For example, in the hilly and mountainous environments in Sichuan province where we have conducted our research for over a decade, the intermediate host snail lives in the irrigation networks that line agricultural fields and are genetically different from those in the provinces along the lower Yangtze River [3], farmers are the predominant populations at risk of infection, and in some areas, few animals contribute to the transmission cycle [4]. These conditions differ markedly from the transmission environment in the lakes and marshland regions of China where infection is strongly associated with fishing, snails live in the marshlands, and bovines are largely responsible for transmission [5]. This may explain why the new national program has been implemented in only five of the seven endemic provinces [2] and suggests that regional differences in schistosomiasis transmission warrant a more flexible approach.

In conducting research over the past two decades in Sichuan, we have also seen evidence of the rebound in infection following chemotherapy programs. From 1987 to 1995 in Xichang county, Sichuan, an intense chemotherapy-based program resulted in reductions in *Schistosoma japonicum* prevalence from 63% to 8% [6]. At the conclusion of this program, the region was left to the lesser resources of county health authorities, who provided limited access to praziquantel chemotherapy. Our work in 2000 in 20 villages in this region showed that infection levels had rebounded to an average prevalence of 29%, with prevalence as high as 73% in one village [4]. Despite administering treatment to everyone who was infected in 2000, prevalence had returned to 68% of previous levels by 2002 in the ten high infection prevalence villages that were followed [7].

Alternatives to largely chemotherapy-based efforts can be designed through careful identification of the individual and environmental drivers of schistosomiasis transmission. In Sichuan, epidemiologic studies and mathematical modeling results indicate that effective, sustainable control requires a combination of chemotherapy, snail control, and improvements in sanitation [8]. Some of the interventions called for in Poyang Lake, such as cattle removal, have questionable social and economic impacts, and would be unlikely to impact transmission in some endemic areas of Sichuan province like Xichang county, where bovine populations are small in number and play a marginal role in transmission [4], though in other parts of Sichuan, bovines may be large infection reservoirs [9]. In contrast, snail control, which was absent from the Poyang Lake intervention, and poses a challenge due to the large marshland area, may play an important role in reducing transmission in Sichuan. This suggests the need for regionally flexible control strategies driven by local circumstances and data.

Second, we question how the considerable cost of comprehensive control programs (approximately US$66,000 per village in the Poyang Lake study) may be best financed and coordinated. If control is to be comprehensive and sustainable, it can only be made possible from relatively large investments in environmental modification and rural sanitation. These capital-intensive efforts can be facilitated by leveraging existing inter-sectoral funding, such as that for rural energy development, and directing it to schistosomiasis-endemic areas that could benefit greatly from household-level biogas sanitation systems [10]. Agencies outside of the Ministry of Health have a key role to play in formulating effective comprehensive control, including those that govern rural sanitation, and energy, water, agricultural, and animal resources. Indeed, it will be important to evaluate whether the new national control program is an effective mandate for inter-sectoral cooperation, and whether such cooperation may be leveraged for high-cost initiatives (e.g., replacing cattle with mechanized equipment, widespread provision of latrines, and concretization of ditches).

Even with commitments across important sectors, the new national control program would be strengthened if it included guidance for moving beyond basic transmission control to regional elimination. In Sichuan, collaborative research with the Sichuan Center for Disease Control and Prevention has led research findings related to the importance of comprehensive control to be quickly integrated into public health practice in the province. In 2008, Sichuan province reached the important milestone of transmission control in all 5,141 of its formerly endemic administrative villages. While this should be celebrated as a tremendous achievement, we are guarded in our enthusiasm, as the threat of re-emergence remains a concern [11].

In fact, there is still much to be learned regarding schistosomiasis elimination and the prevention of re-emergence. Although there is active work on vaccines, particularly for animal reservoirs [12], [13], a vaccine for human schistosomiasis is not yet available. Hence, we remain focused on documenting the environmental determinants of schistosomiasis transmission and the impact of disease control strategies on altering the potential for schistosomiasis transmission and re-emergence. Ongoing research is focused on the development of new methods for identifying reservoirs and pathways for re-emergence, including population genetics approaches utilizing microsatellite analysis of the genome of schistosome larvae [14], and geospatial and modeling methods to understand hydrological and social factors that may be associated with the regional persistence of the parasite in highly connected environments [15]. Ultimately, folding the findings from this region into national Ministry of Health policy may hasten China's move toward schistosomiasis elimination.

## Response by Long-De Wang, Tian-Ping Wang, Hong-Gen Chen, Xing-Qi Dong, Li-Ying Wang, Yang Hao, Robert Bergquist, and Xiao-Nong Zhou: Comprehensive Strategy to Effectively Eliminate the Burden of Schistosomiasis in China, both in the Mountainous and Lake Regions

The sustainable implementation of the current control strategy has always been, and continues to be, an important issue in the Chinese national schistosomiasis control program [16], [17]. Seto et al. have raised three questions concerning the new comprehensive strategy for schistosomiasis control in China, announced by Wang et al., published in early 2009 in the *New England Journal of Medicine* [2]. First, they voiced concerns regarding the wider validity of the study results and the costs associated with the implementation of the control program [4], [5]. Specifically, they questioned how a new national schistosomiasis control strategy could be based on research findings from only four pilot villages, and worried about the considerable costs associated with the implementation of the control program, particularly if it were to be extended from the lake region into the mountainous region. Second, they highlighted that, according to their own experience in the mountainous region of the Sichuan province [6], [7], [8], a comprehensive control strategy could only succeed based on the principle of inter-sectoral collaboration. Finally, they put forward a list of research needs. These questions touch the core of the control program in the lake region and its future expansion to the mountainous region, and we would like to offer the following points for clarification and further discussion.

We appreciate the attention that Seto et al. have paid to our new national schistosomiasis control program and their critique of its individual component in light of their own extensive experience. Here, we would like to respond to two issues, namely (i) how the new comprehensive strategy was translated from the research and pilot testing phase into a control program, and (ii) how a sustainable and efficient funding and management of the control program is envisaged.

Regarding the first discussion point, we would like to provide some additional background information to clarify that issues of scaling-up and strategy adaptation to different environments were considered early on. Basically, in order to successfully transfer a newly developed strategy into a functioning control program, three steps are required. First, a pilot study is undertaken to accumulate field-based evidence regarding the efficiency and feasibility of the new strategy that can be presented to policy makers. This is normally performed on a small scale and in a tightly controlled setting with an aim of generating evidence regarding the efficiency and feasibility of the new strategy. Second, the experiences gained in the pilot study are employed to further revise the strategy, and the new approach implemented in the frame of an existing control program, within various settings and on a larger scale. If successful, the new strategy can then be further scaled up and formally established as the national control program [18].

In line with this three-step principle, a first pilot study of the new comprehensive schistosomiasis control strategy was implemented in two villages in Jinxian county, Jiangxi province, in 2005. In this area, the lake ecosystem is present, and a significant reduction of the human *S. japonicum* prevalence was achieved [2]. Subsequently, the strategy was implemented in another three pilot counties, i.e., in Anxiang county of Hunan province, Hanchuan county of Hubei province, and Guichi county of Anhui province, covering a total of 149,740 inhabitants in the lake region. Results from those pilot counties showed that the human reduction rate was decreased by 87%, 78%, and 61%, respectively, after the intervention had lasted for 3 years. Unfortunately, the results pertaining to those pilot studies were exclusively reported in the Chinese scientific literature, severely limiting their accessibility for international scholars [19], [20], [21]. After accumulating such first-hand experience and further improving the strategy in light of the gained experiences, the strategy was applied in 90 counties in all five endemic provinces in the lake region. Sentinel monitoring has been ongoing in 32 villages in the five provinces since 2007 [22]. At the same time, pilot studies with the new comprehensive control strategy were also implemented in the mountainous regions of two further endemic provinces, i.e., in Penshan county of Sichuan province and in Eryuan county of Yunnan province. In both areas, similar results regarding prevalence reduction as achieved in the lake region were recorded [23], [24], [25]. Thus, a well-planned process of scaling-up had preceded the announcement of the new comprehensive schistosomiasis control strategy in China by labeling the publication of National Regulation of Schistosomiasis Control [21]. We are well aware that the ecosystem, socioeconomic situation, culture, customs, and transmission patterns of *S. japonicum* in the mountainous regions of Sichuan and Yunnan provinces differ significantly from those in the lake region [16], [26], [27], and hence further information is needed as to whether the strategy requires further adaption to better fit local conditions in all areas. It is conceivable that no strategy equally fits all areas.

With regard to the second point, we thank Seto et al. for pointing out that inter-sectoral collaboration is the key for the success of the control program in the mountainous region, indeed, in all endemic areas. We fully agree with this statement based on the available experience gained in the lake region of Jiangxi province. In this are,a the interventions were led and coordinated by the local health departments, either at the provincial or at the county level, and in collaboration with several departments, i.e., those in charge of agriculture, water conservation, forestry, natural resources, communication, and education [22], [28]. Moreover, the new control strategy is also integrated with other programs, commonly subsumed under the slogan “creating a new countryside” [29], [30]. Thus, interventions profited significantly from synergies with other programs, resulting in enhanced outcomes and reduced costs [31]. Therefore, in addition to the reduction of *S. japonicum* prevalence to less than 1% [2] and significantly reduced soil-transmitted helminth prevalences [21], local residents also profited from broader socioeconomic development of their communities.

There is no doubt that the new comprehensive schistosomiasis control strategy in China, focusing on reducing the transmission of *S. japonicum* from cattle and humans to snails, is highly effective. These interventions have been adopted as the national strategy to control schistosomiasis japonica in China after a well-planned strategic process involving a succession of pilot studies, followed by scaling-up [18], [21]. However, there still remains a need to improve cost-effectiveness by different combinations of approaches used to support the sustainable implementation of the national control program. We are convinced that insights gained while addressing the research needs identified by Seto et al. will lead to novel and innovative approaches for the interruption of *S. japonicum* transmission [2], [32].

With the comprehensive strategy applied for 4 years in China, significant reduction of schistosomiasis burden in China has been achieved by scaling-up first-hand experience [19]. China is well on its way toward the goal of reducing the schistosomiasis prevalence to less than 1% both in humans and livestock by 2015 [18]. Field data have showed that the comprehensive strategy can be applied in whole endemic areas with different settings along with some adaption by combination of various approaches, which will lead to the elimination of schistosomiasis transmission in China in the future [32].

## Response by Edmund Seto, Justin Remais, Elizabeth Carlton, Shuo Wang, Song Liang, Paul Brindley, Dongchuan Qiu, and Robert Spear: Common Lessons from Comprehensive Schistosomiasis Control Strategies in China

We thank Wang et al. for elaborating on their study in Poyang Lake [2], and for pointing to additional evidence from the lake environments in Anhui, Hunan, and Hubei and the mountainous environments in Sichuan and Yunnan provinces. The process of scaling up from pilot studies to larger scale control programs and then to national-level schistosomiasis control policy is sensible. All parties appear to agree on the importance of comprehensive control strategies that go beyond chemotherapy, as well as the need for cost-effective programs subject to ongoing monitoring and evaluation. We appreciate the authors' commitment to regional adaptations of the national policy, which may offer efficiencies not possible through a one-size-fits-all approach.

Wang et al. state above that “China is well on her way towards the goal of reducing schistosomiasis burden to less than 1% of the human infection rate by 2015”. Reaching this target would be a tremendous achievement. However, the true test will be whether this level of control can be sustained and ultimately translated into elimination. Vigorous surveillance in controlled areas would provide the long-term data needed to properly estimate cost-effectiveness of regional implementations of China's national program, particularly where routine chemotherapy is terminated and the risk (and cost) of re-emergence must be included. The limited data available in such settings suggest that long-term planning, infrastructure investments, and inter-sectoral collaborations are highly worthwhile [32].

Yet, evaluating programs in areas of low endemicity is currently hindered by current surveillance methods and diagnostic tools [9], [33]. The development of highly sensitive environmental and clinical diagnostics must feature prominently in the national elimination strategy. Similarly, careful investigation of areas where schistosomiasis has re-emerged can inform post-control surveillance strategies, leading to more efficient interventions that accelerate China toward the elimination of schistosomiasis.

Having refined a national control strategy over decades, China's program can serve as a model for highly targeted, evidence-based programs in other countries [16]. China's commitment to comprehensive control strategies, surveillance, and focused research has been the foundation of success to date, and will be the key to schistosomiasis elimination in the future.
